# Electrosprayed Nanosystems of Carbamazepine – PVP K30 for Enhancing Its Pharmacologic Effects

**Published:** 2018

**Authors:** Deniz Abedinoghli, Mohammad Charkhpour, Karim Osouli-Bostanabad, Sevil Selselehjonban, Shahram Emami, Mohammad Barzegar-Jalali, Khosro Adibkia

**Affiliations:** a *Research Center for Pharmaceutical Nanotechnology, Biomedicine Institute, Tabriz University of Medical Sciences, Tabriz, Iran. *; b *Students Research Committee,* *Tabriz University of Medical Sciences, Tabriz, Iran.*; c *Department of Pharmacology and Toxicology, Faculty of Pharmacy, Tabriz University of Medical Sciences, Tabriz, Iran. *; d *Department of Pharmaceutics, Faculty of Pharmacy, Tabriz University of Medical Sciences, Tabriz, Iran.*; e *Drug Applied Research Center, Tabriz University of Medical Sciences, Tabriz, Iran.*

**Keywords:** Carbamazepine, PVP K30, Electrospray, Nanobeads, *In-vivo* evaluation

## Abstract

This study was conducted to enhance the pharmacologic effect of carbamazepine (CBZ) (as a poorly water-soluble drug) by fabricating CBZ-PVP K30 nanobeads using an electrospraying technique. CBZ-PVP K30 nanosystems with various ratios (1:3 and 1:5) at total solution concentrations of 3% and 5% w/v were prepared. The solution concentration extremely affected the size of the samples; where, the nanobeads (mean diameter of 457.65 ± 113.72 nm and 1.16 ± 0.46 µm) were developed at low and high solution concentrations, respectively. DSC thermographs and PXRD patterns precisely showed CBZ amorphization in the electrosprayed nanosystems. Based on the FTIR spectrum of the electrosprayed samples, a feasible interaction between N–H/O–H group of CBZ and PVP carbonyl group was detected. The *in-vitro* release studies revealed that the electrosprayed nanosystems represent a comparable rapid dissolution rate with respect to the physical mixtures (PMs) and the pure drug. The *in-vivo* results in NMRI mice indicated that the electrosprayed nanoformulation (with the drug: polymer ratio of 1:5 at a total solution concentration of 5% (w/v)) prolonged seizure latency time and decreased mortality percent in strychnine (STR) induced seizure mice more efficiently than the PM. Our finding revealed that the electrospraying as a cost-benefit and one step technique could be effectively applied for improving the physicochemical characteristics and pharmacologic effect of CBZ.

## Introduction

Epilepsy is one of the chronic neurological disorders occurring unpredictably and frequently in normal brains that can be characterized by unprovoked seizures, recurrent, and/or brain alterations together with a single seizure which increases the future seizures occurrence potential (1). Mainly excessive, abnormal or hypersynchronous neuronal activities trigger epileptic seizures in the brain (2). About 50 million people with epilepsy are diagnosed globally that near 90% of these patents are recognized in developing countries. Antiepileptic drugs (acetazolamide, clobazam, carbamazepine, eslicarbazepine acetate, clonazepam and *etc.*) are treatments that mainly used to successfully control symptoms of epilepsy.

Carbamazepine (*5H*-dibenz (b,f)azepine-5-carboxamide) (CBZ), is a commonly used antiepileptic drug which has been considered as a bestselling anticonvulsant because of its good therapeutic behavior. The typical CBZ tablets usually administered orally due to having a high intestinal permeability with variable plasma concentrations (4-32 h). The delayed and irregular absorption of CBZ in the body is attributed to its poor water solubility (3, 4). There is a serious problem in drug industries that a major part of newly developed active pharmaceutical ingredients (APIs) have poor solubility in water. These APIs with low solubility and high permeability are categorized as class-II drugs in the biopharmaceutical classification system (5). CBZ is a class-II drug and its absorption characteristics from the gastrointestinal tract (GIT) can be expressed as a dissolution rate limited process. Poor water solubility of a drug usually can result in fed/fasted variations and a poor bioavailability (4, 6), where the low dissolution rate of class-II APIs can be enhanced by particles size reduction (3) due to the effective surface area augmentation of drugs. Hence, CBZ dissolution rate enhancement can extend its absorption after oral administration and improve its bioavailability. Some other strategies for enhancing the dissolution rate of class-II drugs are formulating APIs using polymers (7), surfactants (8), liposomes, and lipids (9). Various methods have been benefited to enhance dissolution rate of CBZ including cocrystallization (10, 11), cogrinding (12), solid dispersion (11), self-microemulsifying (13), adsorption of drugs onto silica substrates (14), nanodispersion (15), and nanoparticles (1, 16). To the best of our knowledge, there is no report regarding the preparation of CBZ electrosprayed nanosystems.

Electrospraying (ES)/electrospinning is a versatile method with capability of manufacturing a variety of micro-nano sized specimens for a wide application range in the pharmaceutical industry (17). The easily modified, economic and one-step technique has been utilized recently with good outputs in many areas such as protein delivery (18), anti-cancer therapy (19), ocular drug delivery (20), and transdermal delivery (21). The principle of ES procedure is based on polymers solution atomization using an electrical power; which a liquid jetted out (atomized) from a liquid filled capillary nozzle (syringe) by imposing a high voltage electrical force to create nano sized formulations (22, 23). Process parameters (formulation flow rate, deposition distance and applied voltage), jetting behavior and the liquid physical characteristics (electrical conductivity, surface tension, density, viscosity and polymer to drug ratios) alteration and controlling, predominantly enable nanofibers or nanoparticles (nanobeads) syntheses (24, 25). Then, the samples can be collected onto a grounded screen/counter electrode placed under the tip of the syringe. Some drugs, such as propranolol hydrochloride (22), naproxen (26), triamcinolone acetonide (24), azithromycin (27), methylprednisolone acetate (28), atorvastatin calcium/ezetimibe (7), and clarithromycin (29) have been successfully processed using this method.

Solubility and bioavailability enhancement of drugs with low aqueous solubility can be attained simply by nanonization alone. However, it is expected that a combination of polymer encapsulation and nanonization can lead to formulations with further solubility improvement. Thus, in the present study polyvinylpyrrolidone (PVP), a food, and drug administration (FDA) approved inactive polymer was used to prepare CBZ–PVP K30 nanoformulations conducting the ES method. PVP as an excipient is extensively used in drug formulations due to its chemically inert, non-toxic, colorless, and non-ionic nature (16). Having universal solubility in various solvents (hydrophobic /hydrophilic) is one of the special characteristics of PVP systems (3, 16). Therefore, CBZ–PVP K30 nanobeads were formulated with different drug to polymer ratios at various solution concentrations to enhance physicochemical characteristics and pharmacological effect of CBZ. Furthermore, pharmacological effects of the prepared nanosystems were investigated in NMRI male mice.

## Experimental

*Materials*

Polyvinylpyrrolidone (PVP K30) and carbamazepine were purchased from Degussa (Darmstadat, Germany) and Arasto Pharmaceutical Chemicals Inc. (Tehran, Iran), respectively. Sodium hydroxide, potassium phosphate monobasic, and ethanol were obtained from Merck (Germany). All other chemical materials were analytical grade.

*Electrospraying procedure*

A custom-designed electrospraying device (Fanavaran Nano-Meghyas, Tehran, Iran) was conducted to prepare CBZ-PVP K30 nanosystems. Briefly, CBZ-PVP K30 solutions with 1:3 and 1:5 drug: polymer ratios were prepared by co-dissolving CBZ and PVP K30 in ethanol at room temperature. The drug: polymer solution total concentrations were regulated to be 3 and 5% (w/v).

The liquid jet of prepared solutions was made by using a voltage of 25 kV applied to the syringe needle (gauge 29) connected to a ring shaped capillary tube made of polyethylene with inner diameter of 0.1 mm. The prepared solutions were jetted towards a polytetrafluoroethylene coated, grounded collector screen to form CBZ-PVP K30 nanobeads. The injection rate and distance between the grounded screen and nozzle tip were fixed at 5 mL/h and 20 cm, respectively.

*Field emission scanning electron microscopy (FE-SEM)*

The Field Emission Scanning Electron Microscope (MIRA3, Tescan Co., Brno, Czech) operating at 20 kV was used to evaluate morphology of the samples. Before analyzing, the electrosprayed samples were coated with a thin gold layer (about 150 Å in thickness) using gold sputtering apparatus (Emitech K550, Kent, UK). 

*Differential scanning calorimetry (DSC)*

The thermal behaviors and thermograms of pure CBZ, PVP K30, physical mixture (PM) and electrosprayed nanosystems were examined by DSC 60 (Shimadzu, Kyoto, Japan). Briefly, the thermal behavior of meticulously weighed (5 mg) samples in sealed aluminum pans were evaluated at a scan rate of 20 °C/min (25-220 °C) and analyzed with TA60 software. The indium and aluminum oxide powders were benefited as standard and reference samples, respectively. 

*Powder X-ray diffraction (PXRD)*

PXRD patterns and crystallinity identification of the pure CBZ, PVP K30, PM, and electrosprayed samples were carried out using X-ray Diffractometer D5000 (Siemens, Munich, Germany) at scanning rate, step size, and 2 θ angle range of 0.6 °/min, 0.02°, and 5°-35°, respectively by K_α_ radiation of Cu (λ = 1.5405 Å) at 30 mA, 40 kV.

*Fourier transform infrared spectroscopy (FTIR)*

The FTIR Spectrophotometer (Shimadzu 43000, Kyoto, Japan) was employed to validate the feasibility of drug-polymer chemical interactions. Briefly, a compact disc of pure CBZ, PVP K30, CBZ-PVP K30 PM and their electrosprayed samples were fabricated by KBr disk technique and evaluated at scanning range of 4000-400 cm^-1^ with average spectra of 32 scans at a resolution of 2 cm-^1^.

*In-vitro drug release*

The dissolution behaviors of pure CBZ, PM, and electrosprayed samples were assessed using USP Apparatus II (paddle method). Samples equivalent to 20 mg of CBZ under rotational mixing (50 rpm) were placed in the vessels with 150 mL of phosphate buffer (pH 6.8) at 37 ± 0.2 °C. At predetermined intervals (5, 10, 20, 30, 45, 60, 90, and 120 min), 1 mL of the processed solutions was carried away and replaced with equal volume of fresh buffer in order to retain a constant dissolution medium. The cellulose acetate membrane (pore size 20 nm, Whatman, Kent, UK) was used to filter the removed solution. To estimate the drug cumulative release graphs, UV spectrophotometer (Shimadzu, Kyoto, Japan) at wavelength of 283.8 nm was conducted. The average values of three assessments were used.

*Animal study*

*Animals*

A total of 60 male NMRI mice (25-30 g) were supplied by Animal Center Laboratory, Pasteur Institute, Iran. The animals were housed under specific conditions of 12 -12 h light to dark cycle in an air conditioned room at 25 ± 2 with a relative humidity of 50 ± 10%. Food (UAR, Villemoissonsur Orge, France) and water were supplied *ad libitum*. All animal procedures were performed according to the ‘Guide for the Care and Use of Laboratory Animals’ of the research center for Laboratory Animal of Tabriz University of Medical Sciences which is in accordance with the National Institutes of Health guidelines (revised 2011) and was approved by the local authorities of Animal Ethics Committees (AEC reference number: IR.TBZMED.REC.1395.1338).

*In-vivo procedure*

Healthy adult mice were randomly allocated into 6 groups consisting of 10 mice each. In order to investigate the antiseizure effect of electrosprayed CBZ-PVP K30 nanoformulations, the mice in all groups were injected intraperitoneally (IP) to induce seizure using strychnine (STR) (3 mg/kg). Before the IP injection of STR, the animals in group I (ES + 0.5 mL sterile saline (Sal)), group III (ES+Sal), and group V (ES+Sal) were orally gavaged with the CBZ-PVP K30 electrosprayed nanoformulations (100 mg/kg; CBZ) for 30 min, 1 h, and 2 h, respectively. The drug: polymer ratio of 1:5 at the total solution concentration of 5% w/v was used as electrosprayed nanoformulations. The mice in group II (PM+Sal), group IV (PM+Sal), and group VI (PM+Sal) were gavaged by the physical mixture (100 mL/kg; CBZ) of CBZ-PVP K30 the same as the ES treated groups and then were injected STR (3 mg/kg; IP) in the predetermined intervals. In other words, this experiment was conducted using two kinds of treated mice, briefly, all animals in groups I, III, and V were received the ES nanoformulations 30 min, 1 h, and 2 h, respectively, before IP injection of STR, where the animals in groups II, IV, and VI were treated with the PM at the same intervals. CBZ-PVP K30 electrosprayed samples antiseizure effect was compared with that of the PM. In this regard, the hind limb tonic extension (HLTE) beginning time and mortality percent in all experimented groups were controlled for 30 min after IP injection of STR.

*Statistical analysis*

Mann Whitney- U and Fisher›s exact tests were applied to compare the groups. Statistical analysis was implemented using Sigmaplot V12 and any variations among the groups were assumed significant at *p* < 0.05 levels. The *in-vivo* data were asserted as mean ± SEM and attained from 10 experimented mice.

## Results and Discussion

*Morphological evaluation of electrosprayed samples*

**Figure 1:**
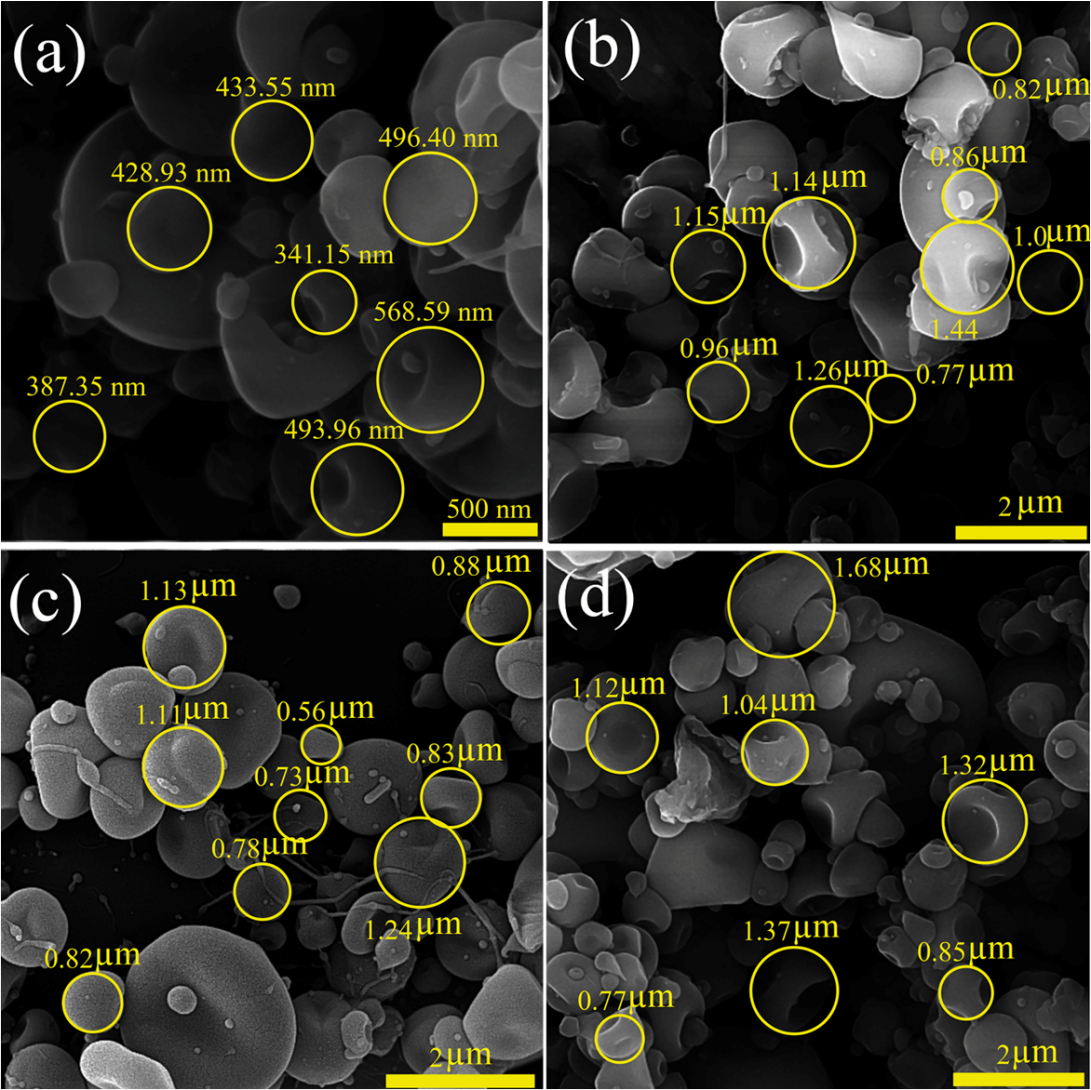
FE-SEM images of the carbamazepine – PVP K30 electrosprayed nanosystems with (a) drug: polymer ratio of 1:3 at the total solution concentration of 3% w/v (Magnification: ×50 k), (b) 1:3-5% w/v (×17 k), (c) 1:5-3% w/v (×15 k) and (d) 1:5-5% w/v (×17 k).

The morphological characteristics and size distribution of the drug particles are critical parameters affecting drug delivery mechanisms and drug effectiveness. Parameters related to the polymer (the polymer type, its concentration, diffusion rate, chains intermolecular interlocking), solvent properties (solvent evaporation rate and its coulomb forces) and working conditions (operating voltage, the prepared solution feeding rate, distance of the nozzle and grounded surface) are the vital factors that can play significant role in the morphological characteristics of the electrosprayed samples (7, 20, 22 and 25). The morphology and size distribution of CBZ-PVP K30 electrosprayed nanoformulations are shown in Figure 1.

Through the current study, except the ratio of the drug: polymer (1:3 and 1:5) and the total concentrations (3 and 5% w/v) of formulated solutions, all the above mentioned parameters were kept fixed. As it is clear from the FE-SEM results, in all formulations the nanobeads in concave shape were formed, so that the average particles size of formulation with drug: polymer ratio of 1:3 at the total solution concentration of 3% w/v was 457.65 ± 113.72 nm (Figure 1a) and the corresponding average particles size of formulations with 1:3 at 5% w/v, 1:5 at 3 and 5% w/v were 0.898 ± 0.34 µm, 1.08 ± 0.34 µm, and 1.16 ± 0.46 µm, respectively (Figures 1b-d). Literature reviewing revealed that high surface tension of the solutions led to the liquid jet dispersion to separate droplets and consequently nanobeads formation. Additionally, the development of the larger nanobeads by enhancing the drug: polymer ratio could be related to electrical conductivity reduction of the prepared solution at the high polymer ratios (7, 22, 28, 30 and 31). PVP electrospuned nanosystems have previously been manufactured and used in some areas such as antimicrobial activity (32), ocular drug delivery (23, 33), and co-drug delivery (7). While to the best of our knowledge, there was no report regarding CBZ-loaded PVP formulations preparation using ES method. 

*Differential scanning calorimetry*

**Figure 2:**
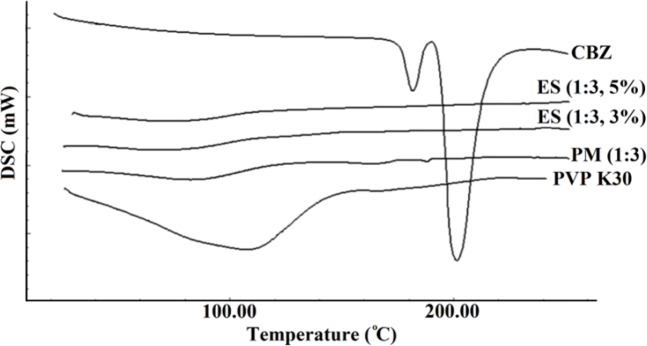
DSC thermograms of the pure carbamazepine (CBZ), PVP K30, physical mixture (PM) and electrosprayed nanosystems (ES) with the drug: polymer ratio of 1:3 at total solution concentrations of 3% and 5% (w/v).

The thermal behavior of pure CBZ, PVP K30, PM, and electrosprayed nanoformulations were examined by DSC (Figure 2). In addition to a versatile co-crystalline, solvated, nanocrystalline, and salt forms of CBZ (3, 10 and 16), various polymorphic of CBZ in solid state forms, have been widely investigated and its five forms including CBZ I, II, III, IV, and V have been reported. CBZ III as a monoclinic structure of this drug is thermodynamically stable form at ambient temperature (15, 34). The only acceptable polymorphic forms of CBZ by the European Pharmacopoeia is CBZ III (35). Two characteristic endothermic peaks at 178 °C and 195 °C attributed to melting points of CBZ III and I forms, respectively. As it is clear one exothermic peak separated these two endothermic peaks at 180 °C which corresponds to recrystallization of CBZ polymorphic form I from CBZ III melt. This thermogram confirms that the used pure CBZ in this study contains CBZ III polymorphic form (3, 35).

Scanning of PVP K30 revealed a broad endothermic peak ranging from 75 to 140 °C, illustrating water loss of this polymer because of PVP polymers hygroscopic nature (7). The corresponding melting endotherms were not detectable in the thermograms of CBZ-PVP K30 physical mixture and electrosprayed samples indicating crystallinity reduction of CBZ (7, 36). Based on literature review, the drugs’ structure during ES procedure could transform from a crystalline state to an amorphous form (3, 22, 28 and 31) and this amorphous structure formation as well as PVP inhibitory effect on the drug crystallization in the ES samples have been reported in our previous work and other studies (7, 36).

*Powder X-ray diffraction (PXRD) evaluation*

**Figure 3:**
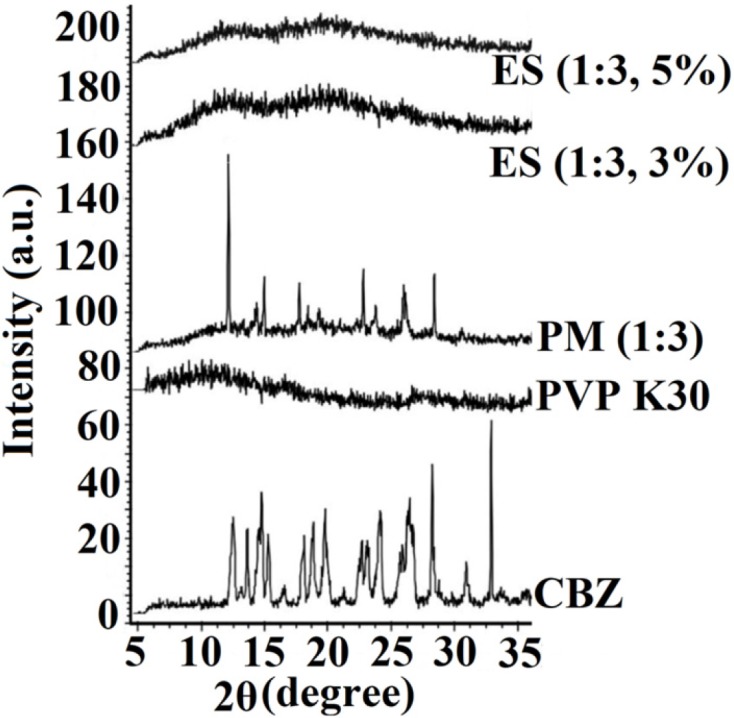
PXRD patterns of the pure carbamazepine (CBZ), PVP K30, physical mixture (PM) and electrosprayed nanosystems (ES) with the drug: polymer ratio of 1:3 at total solution concentrations of 3% and 5% (w/v).

The crystallinity of the pure drug, PVP K30, PM, and electrosprayed samples were identified using X-ray diffractometer (Figure 3). According to USP Reference Standard and International Center for Diffraction Data; the sharp, distinctive diffraction peaks at 2 θ angles of 13.15°, 14.25°, 15.36°, 15.9°, 19.55°, 23.45°, 25.0°, and 27.7° demonstrated the crystalline characteristics of pure CBZ that is in good agreement with previous studies with regard to CBZ III polymorphic form (37).

The absence of any characteristic peaks in the PXRD curve of PVP K30 showed its amorphous behavior. The PXRD pattern of PM showed the characteristic peaks of CBZ with a reduced intensity due to the possible dilution effect of PVP. Although, CBZ preserved its crystalline structure in the PM; however, no distinctive diffraction peak was indicated in the PXRD patterns of CBZ-PVP K30 electrosprayed nanosystems, suggesting CBZ transformation to an amorphous form during the preparation process. These results have good consistency with DSC findings and previously reported works on other electrosprayed drugs, such as propranolol hydrochloride (22), naproxen (26), triamcinolone acetonide (24), azithromycin (27), methylprednisolone acetate (28) and atorvastatin calcium/ezetimibe (7).

*Fourier transform infrared spectroscopy*

**Figure 4:**
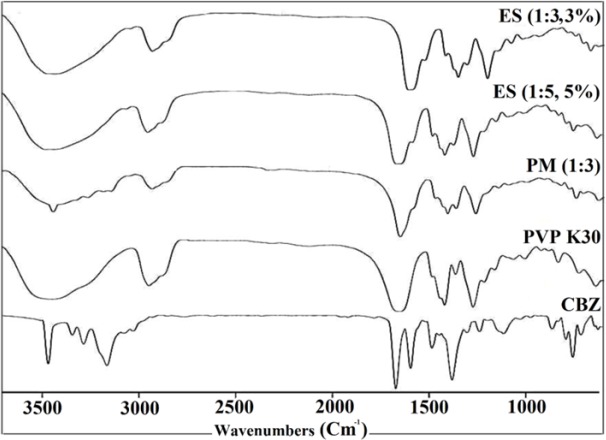
FTIR curves of the pure carbamazepine (CBZ), PVP K30, physical mixture (PM) and electrosprayed nanosystems (ES) with the drug: polymer ratio of 1:3 at total solution concentrations of 3% and 5% (w/v).

FTIR spectroscopy has been applied broadly to detect the nature of possible interactions in polymer mixtures. The basis of conducting an IR spectroscopy to investigate the blends of two components in polymer mixtures is that, at the molecular state these components will cause alterations in the molecules oscillating dipole. These changes can be detected as the bandwidth and frequency changes of the spectrum in the interacting groups. Therefore, CBZ and PVP interactions could be detected by comparing the IR spectra of the electrosprayed samples and PM with that of the pure polymer and drug (shifting and broadening of functional groups in IR spectra will represent any possible interactions) (38). In this regard the FTIR spectrophotometer was applied to detect the possibility of any drug-polymer chemical interactions in the solid state (Figure 4).

The FTIR spectrum of CBZ displayed distinctive peaks at 3466 and 3161cm^-1^ (–NH valence vibration), 1677 cm^-1^ (–CO–R vibration), 1605 and 1595cm^-1^ (range of–C=O– and –C=C vibration and –NH deformation) which is in agreement with previously reported IR spectra of CBZ (3, 36). Moreover, FTIR spectra of pure PVP K30 indicated the peaks at 2916 cm^-1^ (CH aliphatic stretching), 1656 cm^-1^ (-C=O stretching), 1290 cm^-1^ (C–N stretching), and a broad peak due to the water presence between 3200 and 3600 cm^-1^ (in good agreement with the DSC data) (7). The PM infrared spectra showed the drug and polymer characteristic absorption bands, demonstrating presence of CBZ and PVP K30. Additionally, CBZ and PVP K30 typical bands were detectable in the FTIR spectrum of the electrosprayed formulations with reduced intensity, whereas this phenomena could be related to the dilution effect of the polymer (22, 27 and 28). However, as it is clear from the FTIR spectrum of PM and ES samples, the characteristic IR bands of CBZ and PVP K30 at these samples were shifted slightly to lower frequencies, suggesting the probability of hydrogen bonding development between CBZ and PVP K30 carbonyl groups (3, 7). Existence of carbonyl and nitrogen groups on the pyrrole ring of PVP enabled it to form a hydrogen bond. Where, nitrogen atoms involvement in intermolecular interactions could be restricted by steric hindrance, so carbonyl group is more appropriate to form hydrogen bonding (38). Generally, hydrogen bonding as a noncovalent interaction could lead to the peaks broadening or bathochromic shifting of functional groups (7). Thus, by considering CBZ and PVP K30 chemical structures, interactions between N–H or O–H group of CBZ and PVP carbonyl group’s and formation of a hydrogen bonding is more feasible. Other studies containing PVP as the polymer with different drugs including ezetimibe (7), carbamazepine (3), and indomethacin (39) were previously reported same results.

*In-vitro dissolution study*

**Figure 5:**
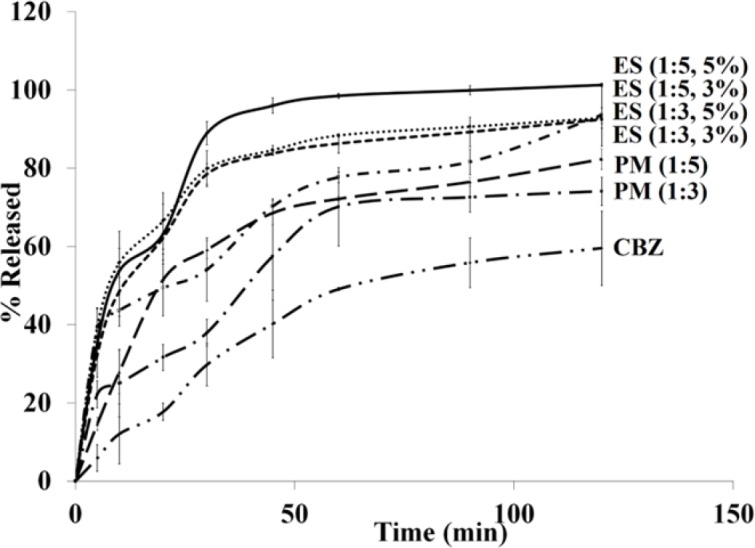
Dissolution profiles of the pure carbamazepine (CBZ), physical mixtures (PM) with drug: polymer ratios of 1:3 and 1:5, and electrosprayed nanosystems (ES) with the drug: polymer ratios of 1:3 and 1:5 at total solution concentrations of 3%, and 5% (w/v).

The drug release profiles of pure CBZ, PMs, and electrosprayed nanosystems were assessed using USP apparatus II (paddle method) (Figure 5). The effect of electrospraying procedure and the polymer ratio on the drug release behavior were evaluated by calculating t_45%_ (corresponding percent of the released drug within 45 min) and DE_120 min_ value (the dissolution efficiency (DE) up to 120 min) are illustrated in Table 1. The area under the drug release profile is called DE calculated up to a determined time (t) and illustrated as a percent of the rectangle area expressed by 100% of drug release at the same time (40). DE can be calculated by Equation (1):

$\mathrm{DE}\left(\%\right)=\frac{\int_{0}^{t}\mathrm{ydt}}{y_{100}t}\times 100$

(1)

Where, y is the dissolved drug percent at time t. Based on the selected intervals, a range of values can be calculated for the DE. However, a specified time interval should be selected in the case of studying a set of data. DE_120 min_ were calculated from the release profiles of the pure drug, PMs, and ES nanoformulations in the present study and benefited for comparison.

**Table 1:** Calculated amounts of the t45% and DE120 min for pure carbamazepine (CBZ), physical mixtures (PM) with drug: polymer ratios of 1:3 and 1:5, and electrosprayed nanosystems (ES) with the drug: polymer ratios of 1:3 and 1:5 at total solution concentrations of 3%and 5% (w/v).

**Sample**	**t**_45 min (%)_	**DE**_120 min_
CBZ	40.19 ± 8.63	40.5
PM 1:3	57.50 ± 8.00	56.5
PM 1:5	68.60 ± 3.02	64.0
ES 1:3, 3%	70.26 ± 1.86	69.0
ES 1:3, 5%	83.73 ± 0.40	78.0
ES 1:5, 3%	84.63 ± 1.19	79.5
ES 1:5, 5%	95.99 ± 1.90	87.0

Considering t_45%_ and DE_120 min_ values at a specified pH, it can be concluded that the electrosprayed nanosystems had meaningfully faster drug release rate than the PMs and pure drug with enhanced dissolution efficiency. So that, t_45 min _= 95.99% and DE_120 min _= 87% were determined for the nanoformulations with the drug: polymer ratio of 1:5 at total solution concentration of 5%, while the corresponding values were estimated to be 40.19% and 40.5% for crystalline CBZ.

The observed rapid drug dissolution rate from electrosprayed nanosystems could be attributed to: a) Drug amorphization during the fabrication process (as witnessed by DSC and PXRD data), thoroughly dispersion of CBZ in the PVP K30 matrix and PVP K30 solubilizing effect, so there is no need to extra forces for overcoming the crystal lattice energy. b) Elevation of the surface area owing to the particle size reduction; thus the drug dissolution rate is improved according to the Noyes-Whitney equation. c) Decrease of the diffusion layer thickness around the formulated drug as a result of size reduction (7, 30, 41 and 42). The saturation solubility augmentation is another significant aspect that has usually been neglected. It is considered a compound-specific constant which depends only on the dissolution medium characteristics and temperature. However, the saturation solubility turned out to be a particles size dependent when the particles size reduces to a size in the range of nanometer. The Kelvin, Ostwald, Freundlich, and Prandtl equation is the theoretical background of this phenomenon (43-45). It has been indicated that the vapor pressure on a curved surface is a function of the curvature (Kelvin equation) and because of strong curvature of a liquid droplets, they indicate an enhanced vapor pressure under a specific size. Consequently, an accelerated phase transformation from a liquid to a gas phase can be occurred in the liquid molecules due to this increased vapor pressure (escaping tendency). This equation identifies the vapor pressure relation with the phase transition of the liquid molecules and can be expanded to drugs dissolution behavior (molecules transition from a solid phase to a liquid phase). In another word, in the Kelvin equation, the dissolution pressure can be considered corresponding to the vapor pressure. In a dissolution medium, a balance between the dissolved (dissolution pressure) and recrystallized molecules determines the saturation solubility. This equilibrium will shift by augmenting the dissolution pressure which, as a result, will increase the saturation solubility. Furthermore, this fact (the relationship between the particle size and the saturation solubility) has been indicated on the above mentioned equation. The dissolution rate enhancement and drug diffusion accelerating are two main result of the increase in saturation solubility. The former could be explained according to the Noyes-Whitney equation and the second is due to the concentration gradient increasment between the blood and lumen by enhancing the saturation solubility in the gut lumen and promoting the drug absorption (43-45).

CBZ is a class-II drug and its absorption characteristics from the GIT can be expressed as a dissolution rate limited. Where, CBZ dissolution rate enhancement can influence its absorption after oral administration and improve its bioavailability. Solubility and consequently bioavailability enhancement of drugs with low aqueous solubility can be attained simply by nanonization alone. However, it is expected that a combination of polymer encapsulation and nanonization can lead to formulations with further solubility improvement. As it is clear from the dissolution curves in Figure 5, all the samples depicted a relatively triphasic release pattern; to be precise, the initial rapid release within the first minutes was followed by the gradual and sustained release subsequently. By considering these release profiles it is clear that applying ES technique, could remarkably increase the dissolution rate of the electrosprayed drug in comparison to the corresponding pure CBZ and PMs. Drug encapsulation on the superficial layers of the particles as well as the high surface area due to the smaller size of the nanobeads are two phenomena which could explain the rapid release. The gradual release could be related to the PVP K30 solubilizing effect; while, the diffusion and dissolution of the drug from inner layers elucidated plateau (sustained) phase (3, 7, 22, 27, 36 and 46). As indicated in FTIR data, there was the probability of hydrogen bonding, development between CBZ and PVP K30 carbonyl groups. Hence, formation of CBZ dihydrate form may be restricted due to this interaction. The dihydrate form of CBZ has higher release rate/solubility compared to CBZ anhydrous form and this form of CBZ usually is developed by exposing the anhydrous CBZ molecules to water (46). This could explain the release behavior of CBZ nanobeads using PVP (3). The fast release rate of the electrosprayed sample with the drug: polymer ratio of 1:5 at total solution concentration of 5% (w/v) made it an appropriate system for the *in-vivo* delivery of CBZ.

*In-vivo study*

About 50 million people with epilepsy are diagnosed globally that near 90% of these people are recognized in developing countries. CBZ (a class-II drug) is a commonly used antiepileptic drug that mainly used to successfully control symptoms of epilepsy (47). In the present study, STR induced seizure mice were used to investigate the antiepileptic effects of CBZ-PVP K30 electrosprayed nanoformulations. STR as a potent convulsant drug and particular inhibitor of glycine receptors, by blocking the motor neurone inhibition feedback using the Renshaw cell, exerts its convulsant effect (48). The mean values of the HLTE initiation time and mortality percent in all ESs and PMs administrated groups, 30 min after IP injection of STR, are given in Table 2.

**Table 2:** Average values of the recorded hind limb tonic extension (HLTE) initiation time and mortality percent for 30 min post strychnine (STR) injection in the experimented mice (n = 10).

**Treatment group**	**Seizure latency (s)**	**Mortality after 30 min (%)**
Group I, STR+ ES (0.5 h)	900.5^***^	10^**^
Group II, STR+ PM (0.5 h)	312.0	80
Group III, STR+ ES (1 h)	772.5^**^	10^*^
Group IV, STR + PM (1 h)	282.5	70
Group V, STR + ES (2 h)	262.5	40
Group VI, STR + PM (2 h)	272.5	80

Statistically significant

*p < 0.05,

**p < 0.01,

***p < 0.001

PM: Physical mixture (drug: polymer ratio of 1:5); ES: Electrosprayed sample with the drug: polymer ratios of 1:5 at total solution concentration of 10% (w/v). The data were reported average ± SEM (standard error of mean).

The results clearly revealed that the mice in the groups treated with ES nanoformulations 30 min (group I) and 1 h (group III) before IP injection of STR significantly postponed HLTE initiation time (*p* <0.001), in comparison, those treated with PM (group II and IV) did not show significant delay in HLTE beginning time. However, there was no significant difference in seizure latency time between the groups received ES nanoformulations (group V) and PM (group VI) in 2 h before STR injection (*p* > 0.05). With regard to the mortality survey, the data showed that mortality rates in mice of groups (I) and (III) significantly decreased (*p* < 0.01 and *p* < 0.05, respectively), where, there was no significant reduction in mortality rates of the other treated groups with ES samples and PM (*p *> 0.05). By considering these results it can be concluded that the observed enhanced drug dissolution rate from electrosprayed nanosystems can be the appropriate reason of this *in-vivo* behavior. In other word, CBZ dissolution rate enhancement might be extended its absorption after oral administration and consequently improve its bioavailability in 0.5 and 1 h before IP injection of STR. Besides, it has been mentioned that rodents like mice and rats rapidly eliminate most drugs (49, 50) and CBZ active metabolite half-life in the rats determined to be 1.2- 3.5 h (51, 52), where the elimination rate of antiepileptic drugs (AEDs) in mice usually is more rapid than in rats (49). Additionally, metabolic tolerance may occur in the mice/rats when using CBZ, because of the enhanced elimination rate of the drug by induction of AED metabolizing enzymes. When the side effects of AEDs are considered, the tolerance is clinically advantageous but when the main focus is the antiepileptic efficacy of drugs it is disadvantageous (53). So, the observed increase in mortality rate and seizure latency reduction in groups (V and VI) treated with electrosprayed nanosystems/PM in 2 h before IP injection of STR could be related to these facts.

## Conclusion

Nanobeads of CBZ (as a poorly water soluble drug) were effectively formulated using electrospraying technique. The microstructure studies revealed that the drug: polymer ratios (1:3 and 1:5) as well as the total solution concentrations variation 3-5% (w/v) particularly affected the size of the nanostructures, where by increasing the solution concentration the beads size increased. DSC and PXRD results showed that the crystalline structure of CBZ was transformed to the amorphous form during the electrospraying procedure. Based on the *in-vitro* drug release tests, the electrosprayed nanosystems depicted meaningfully faster drug release rate than the PMs and pure drug. This could be attributed to the amorphization of CBZ, homogenous dispersion of the drug in the highly hydrophilic polymer and its enhanced surface area due to the smaller size of the prepared nanosystems. Furthermore, according to the *in-vivo* results, the electrosprayed nanoformulations increased the STR induced seizure latency and reduced mortality percent more effective than the PM. Our finding revealed that the electrospraying as an economic and one step technique could be effectively applied for improving the physicochemical characteristics and pharmacologic effect of CBZ. 
